# Effect of TNFα stimulation on expression of kidney risk inflammatory proteins in human umbilical vein endothelial cells cultured in hyperglycemia

**DOI:** 10.1038/s41598-021-90496-w

**Published:** 2021-05-27

**Authors:** Zaipul I. Md Dom, Caterina Pipino, Bozena Krolewski, Kristina O’Neil, Eiichiro Satake, Andrzej S. Krolewski

**Affiliations:** 1 Research Division, Joslin Diabetes Center, Boston, MA USA; 2 Department of Medicine, Harvard Medical School, Boston, MA USA; 3 Department of Medical, Oral and Biotechnological Sciences, Center for Advanced Studies and Technology, University G. D’Annunzio of Chieti-Pescara, Chieti, Italy; 4 Section on Genetics and Epidemiology, Joslin Diabetes Center, One Joslin Place, Boston, MA 02215 USA

**Keywords:** Kidney, Kidney diseases, Biomarkers, Proteomic analysis

## Abstract

We recently identified a kidney risk inflammatory signature (KRIS), comprising 6 TNF receptors (including TNFR1 and TNFR2) and 11 inflammatory proteins. Elevated levels of these proteins in circulation were strongly associated with risk of the development of end-stage kidney disease (ESKD) during 10-year follow-up. It has been hypothesized that elevated levels of these proteins in circulation might reflect (be markers of) systemic exposure to TNFα. In this in vitro study, we examined intracellular and extracellular levels of these proteins in human umbilical vein endothelial cells (HUVECs) exposed to TNFα in the presence of hyperglycemia. KRIS proteins as well as 1300 other proteins were measured using the SOMAscan proteomics platform. Four KRIS proteins (including TNFR1) were down-regulated and only 1 protein (IL18R1) was up-regulated in the extracellular fraction of TNFα-stimulated HUVECs. In the intracellular fraction, one KRIS protein was down-regulated (CCL14) and 1 protein was up-regulated (IL18R1). The levels of other KRIS proteins were not affected by exposure to TNFα. HUVECs exposed to a hyperglycemic and inflammatory environment also showed significant up-regulation of a distinct set of 53 proteins (mainly in extracellular fraction). In our previous study, circulating levels of these proteins were not associated with progression to ESKD in diabetes.

## Introduction

Tumor necrosis factor alpha (TNFα) is a potent pro-inflammatory cytokine that exerts its pleiotropic effects on a wide variety of cell types and plays a vital role in the pathogenesis of inflammatory diseases^1,2^. TNFα is predominantly produced by activated macrophages and monocytes^3^, although other cells are capable of producing it. TNFα mediates its biological activities through its two membrane receptors; TNF receptor 1 (TNFR1 also known as TNF-RSF1A) and TNF receptor 2 (TNFR2 also known as TNF-RSF1B)^4,5^.

Endothelial cells represent key effectors in inflammation and short-term treatment of TNFα-induced endothelial cells resulted in the up-regulation of inflammatory cytokines, including interleukins 6 (IL-6) and 8 (IL-8), and adhesion molecules such as intercellular adhesion molecule-1 (ICAM-1), vascular cell adhesion molecule-1 (VCAM-1) and selectins (e.g. E-selectin)^6,7^. A comprehensive and large-scale proteomic analysis of human endothelial cells by Gautier et al. identified 207 proteins that exhibited a significant variation following TNFα-interferon-gamma (IFNγ) stimulation. That study also deciphered at the proteomic level the biological networks involved in endothelial cell response to TNFα-IFNγ^8^. It is worthwhile to note that prior studies mainly examined the intracellular content of TNFα-induced endothelial cells and targeted only a few genes and/or proteins, and importantly matrices such as supernatants (medium that the cells were grown in) have not been investigated.

The pro-inflammatory cytokine TNFα is believed to be a key inducer and driver of inflammation and plays a central role in the network of pro-inflammatory cytokines contributing to the pathogenesis of diabetic kidney disease (DKD) progression. Many factors including high glucose (hyperglycemia), angiotensin II and advanced glycation end-products (AGEs) serve as potent inducers of TNFα, which upregulates the expression of cell adhesion molecules (ICAM-1 and VCAM-1), monocyte chemoattractant protein 1 and colony-stimulating factor-1 in various kidney compartments, thereby promoting the recruitment of monocytes and macrophages to sites of inflammation^9^. In addition, AGEs bind to their receptor RAGE, and the activation of RAGE by AGEs increases endothelial permeability and causes the release and upregulation of TNFα and other cytokines such IL-6 and IL-8, which consequently induces the production of reactive oxygen species leading to glomerular injury and tubular damage, and ultimately leads to DKD^9^.

Hasegawa et al.^10^ were the first to suggest TNFα may participate in the pathogenesis of DKD. Following this initial report, other experimental works have consistently reported TNFα as a critical mediator in the development of DKD^11,12^ and the roles of TNF pathway in the pathogenesis of DKD and other kidney diseases have also been reviewed^13–15^. Recently, attention has been drawn to determine whether TNFα is a potential target for therapeutic intervention to ameliorate the progression of DKD. One study reported the effectiveness of anti-TNFα antibody in the amelioration of DKD in *Ins2*^*Akita*^ diabetic mice and protection from streptozocin-treated hyperglycemic kidney injury in another macrophage-specific TNFα-deficient mice model. In both models, the authors observed significant reductions in albuminuria, improvement in kidney morphology and down-regulation of inflammatory cytokines, in addition to reductions in TNFα levels^16^.

Recently, we comprehensively examined 194 circulating inflammatory proteins in DKD^17^. We identified a robust kidney risk inflammatory signature (KRIS), comprising 17 circulating inflammatory proteins, including previously identified TNFR1 and TNFR2 receptors, interleukins and chemokines. The KRIS proteins were strongly associated with the 10-year risk of end-stage kidney disease (ESKD). It has been interpreted that elevated levels of these proteins in circulation, particularly TNFR1 and TNFR2 might be secondary to increased level of their TNFα ligand in circulation. Elevated levels of TNFα have been observed in individuals at risk of DKD, although inconsistency still existed with some studies reported TNFα levels had no significant change in diabetic individuals^18–20^. These findings, however, may be qualified. First, TNFα is a low abundance protein, hence, very low concentration in circulation, and the currently available methods for detection of TNFα are not sensitive enough to tackle the challenges of TNFα measurement at lower concentrations. Second, if TNFα levels in circulation are changing over a short period of time, therefore, a single measurement of this cytokine may not be a reliable predictor of progression to ESKD during 10-year follow-up. Finally, TNFα may exert its action without being cleaved off or shed from the cell membrane surface by a disintegrin and metalloproteinase 17 (ADAM17), and subsequently released into the extracellular space and into circulation^21,22^. Considering the above hypotheses, elevated level of TNFα might be a driver of the disease process that underlies progression to ESKD and may be responsible for elevated levels of KRIS proteins in circulation.

To investigate whether TNFα may regulate the expression levels of the KRIS proteins, we performed an in vitro study using endothelial cells as a target for TNFα under high glucose (hyperglycemia) condition. In addition to KRIS proteins, we sought to investigate whether TNFα is involved or regulates the expression of other proteins in both the cell lysate (intracellular) and the supernatant (extracellular) from human umbilical vein endothelial cells (HUVECs), in response to TNFα stimulation and hyperglycemia. Toward these aims, we utilized a global proteomic approach based on an aptamer-based SOMAscan proteomic assay that uses single-stranded DNA aptamers^23,24^. As a model system, we selected HUVECs because they are a widely used cellular approach to study biological mechanisms under controlled conditions^25^. We could, therefore, compare the activities of both endogenous and exogenous protein profiles in hyperglycemia and TNFα-induced HUVECs. In addition to the KRIS proteins, the SOMAscan assay also allowed us to quantify 1305 unique proteins, providing an in-depth proteomic analysis of hyperglycemia and TNFα-stimulated HUVECs and resulting in a much more detailed picture of the proteomic variations associated with the inflammatory response.

## Results

### Cellular studies in HUVECs, RPTECs and fibroblasts

Using the custom-made Olink proteomics platform, we quantified the expression levels (pg/ml) of select KRIS proteins (TNF-R1, TNF-R2, EDA2R and RELT) in the cell lysates and supernatants of 3 human cell lines; HUVECs, renal proximal tubule epithelial cells (RPTECs) and fibroblasts. Of the 3 human cell lines, we detected a considerable amount of KRIS proteins in both the cell lysate and supernatant from HUVECs (Supplementary Fig. S1) and, since we aimed to investigate the intracellular and extracellular protein levels exposed to TNFα in the presence of hyperglycemia, HUVECs were selected as the model system in this study.

### Proteomic data assessment

The experimental study design is depicted in Fig. 1. We comparatively analyzed expression profiles of 1305 proteins measured on the SOMAscan platform in HUVECs cell lysate (intracellular) and supernatant (extracellular) in response to hyperglycemia and TNFα treatment versus hyperglycemia alone condition. For defining proteins that exhibited significantly different expression levels, two significant thresholds were applied to derive confident data sets of proteins: (1) α = 2.9 × 10^−3^ (nominal P-value after Bonferroni’s correction for 17 KRIS proteins measured) and (2) α = 3.8 × 10^−5^ (Bonferroni’s correction for 1305 proteins measured on the SOMAscan platform). The fold change is a ratio of a mean RFU value of a protein in HUVECs cultured in TNFα in hyperglycemia condition to a mean RFU value of a protein in HUVECs cultured in hyperglycemia alone. Interestingly, a comparison of expression profiles of 1305 proteins from HUVECs treated with high glucose (4.5 g/L) versus low glucose (1 g/L) condition showed no substantial impact on the expression protein levels in either the cell lysate or supernatant (Supplementary Fig. S2).

**Figure﻿ 1:**
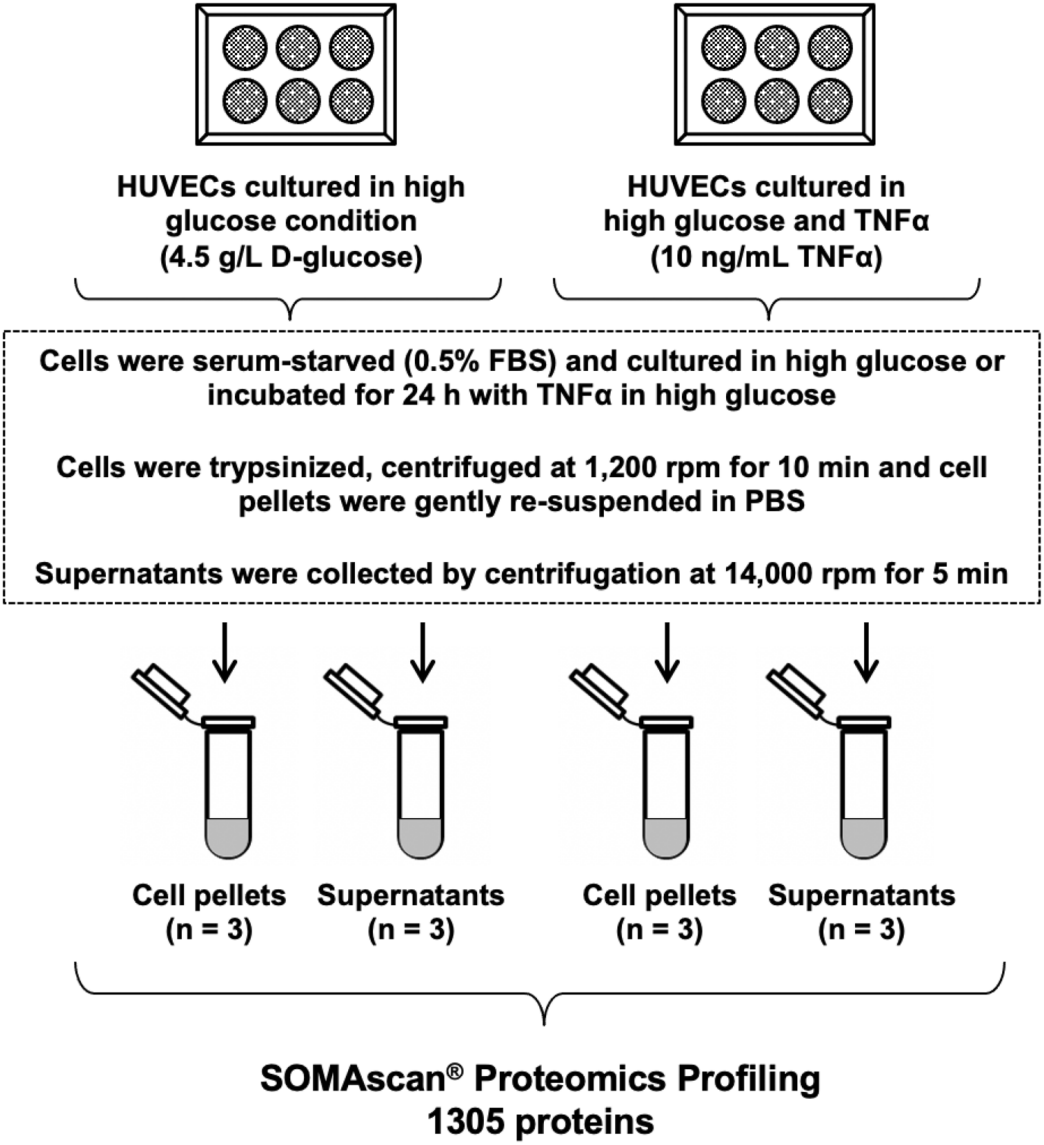
Study design. Experimental study design of the human umbilical vein endothelial cells (HUVECs) treated with high glucose (4.5 g/L D-glucose) alone and with tumor necrosis factor alpha (TNFα; 10 ng/mL) together with high glucose. Each treatment was performed in triplicate.

### Intracellular and extracellular concentrations of KRIS proteins in HUVECs exposed to TNFα and hyperglycemia

All 17 KRIS proteins were present in both intracellular and extracellular HUVECs fractions following TNFα treatment in hyperglycemia condition (Supplementary Table 1). Interestingly, there was very high extracellular TNF receptor superfamily member 21 (TNFRSF21; Mean RFU: 1260 (intracellular) versus 8112 (extracellular)) and TNF ligand superfamily member 15 (TNFSF15; Mean RFU: 928 versus 8210) protein levels in comparison to intracellular protein levels (Supplementary Table 1).

Table 1 shows the list of 17 circulating KRIS proteins associated with risk of ESKD and their fold changes in intracellular and extracellular HUVECs following TNFα treatment in hyperglycemia condition. In the intracellular HUVECs fraction, only IL18R1 was up-regulated whereas CCL14 was down regulated. In the extracellular HUVECs fraction, as expected IL18R1 was up-regulated and 4 KRIS proteins (TNFR1, TNFRSF21, CD300C, CCL14) were down-regulated. Of interest, TNFR2 concentrations were not affected and TNFR1 was profoundly down-regulated following TNFα treatment in hyperglycemia condition. The other KRIS proteins were unaffected by exposure to hyperglycemia and TNFα treatment.

**Table 1:** List of recently identified 17 circulating KRIS proteins associated with risk of development of ESKD (14) and their corresponding intracellular and extracellular protein levels (expressed as fold changes) following TNFα treatment and hyperglycemia condition.

Protein name	Gene symbol	Intracellular		Extracellular	
		Fold change	*p* value	Fold change	*p* value
**TNF-RSF members**					
TNF receptor superfamily member 1A	TNFR1	1.00	n.s	**0.37**	6.2 × 10^−6^
TNF receptor superfamily member 1B	TNFR2	1.06	n.s	0.99	n.s
TNF receptor superfamily member 21	TNFRSF21	0.81	n.s	**0.61**	2.7 × 10^−3^
TNF receptor superfamily member 19	TNFRSF19	0.99	n.s	0.98	n.s
TNF receptor superfamily member 27	EDA2R	1.05	n.s	1.10	n.s
TNF receptor superfamily member 19L	RELT	0.87	n.s	0.91	n.s
**Other proteins**					
Interleukin-15 receptor subunit alpha	IL15RA	1.02	n.s	1.14	n.s
Interleukin-17F	IL17F	1.20	n.s	1.06	n.s
Complement decay-accelerating factor	CD55	1.13	n.s	0.83	n.s
CMRF35-like molecule 6	CD300C	1.01	n.s	**0.92**	2.6 × 10^−3^
TNF ligand superfamily member 15	TNFSF15	1.12	n.s	0.93	n.s
C–C motif chemokine 14	CCL14	**0.61**	8.8 × 10^−5^	**0.15**	1.9 × 10^−5^
C–C motif chemokine 15	CCL15	0.95	n.s	1.01	n.s
Macrophage colony-stimulating factor 1	CSF1	0.93	n.s	1.36	n.s
Hepatitis A virus cellular receptor 2	HAVCR2	1.03	n.s	0.97	n.s
Interleukin-1 receptor type 1	IL1R1	0.98	n.s	1.01	n.s
Interleukin-18 receptor 1	IL18R1	**2.81**	4.5 × 10^−6^	**1.81**	6.8 × 10^−5^

TNF, tumor necrosis factor; TNF-RSF, tumor necrosis factor receptor superfamily; n.s., not significant. Fold changes indicated in bold text indicate differentially expressed proteins at the Bonferroni’s correction α = 2.9 × 10^−3^ (Bonferroni’s correction for 17 KRIS proteins). Fold change is a ratio of a mean RFU concentration of a protein in HUVECs incubated with TNFα in hyperglycemia to a mean RFU concentration of a protein in HUVECs cultured in hyperglycemia.

### Intracellular and extracellular concentrations of other proteins in HUVECs exposed to TNFα and hyperglycemia

**Figure 2:**
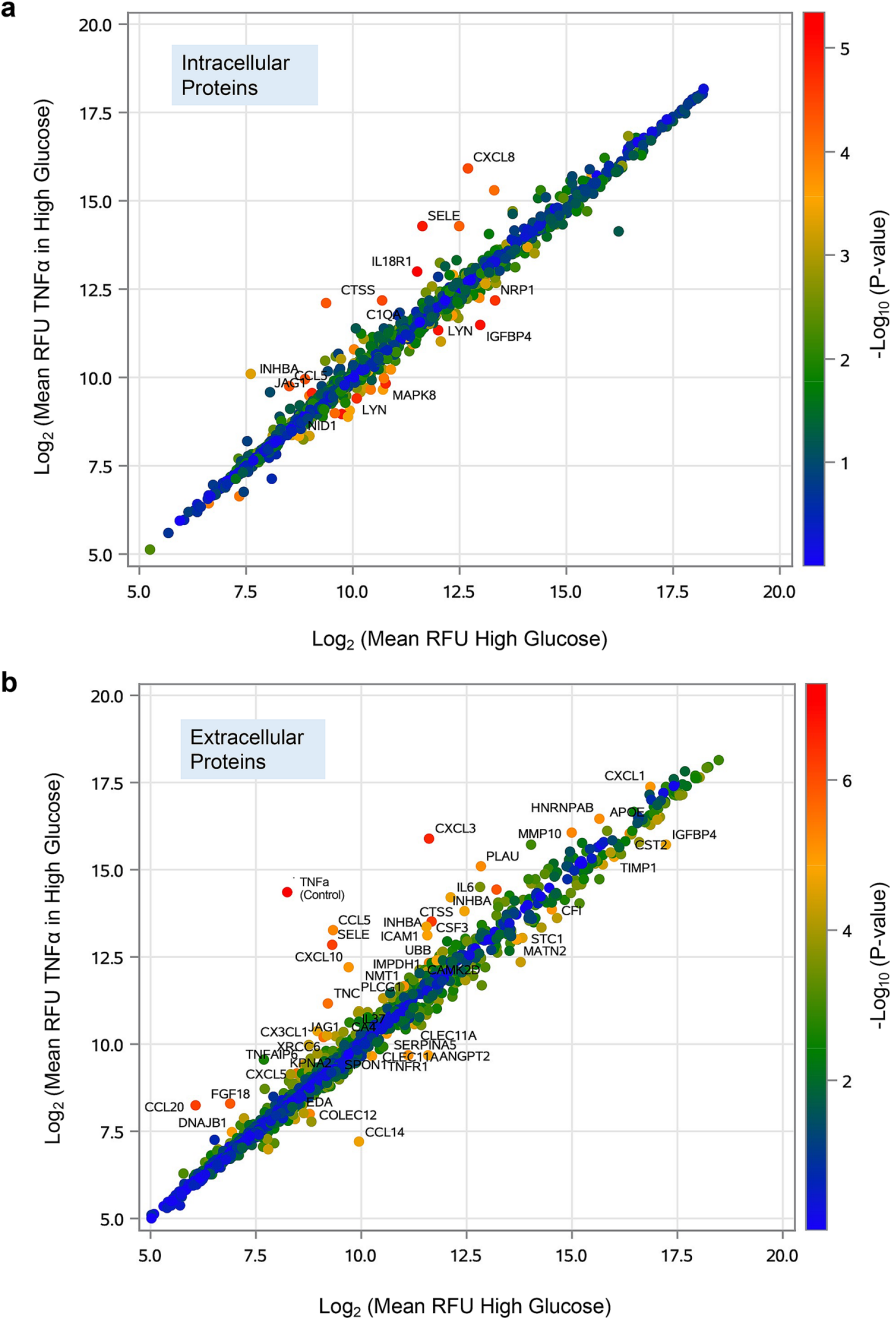
Protein expression profiles in HUVECs exposed to TNFα in hyperglycemia condition vs. hyperglycemia alone. Scatterplots comparing (**a**) intracellular and (**b**) extracellular protein expression profiles in HUVECs exposed to TNFα (10 ng/mL) in high glucose vs. high glucose (4.5 g/L) alone. The values plotted are the mean RFU values (log_2_ scaled for 3 replicates) for the TNF-α in high glucose (*y* axis) and the high glucose (*x* axis) groups. The color of each point indicates the P-values intensity (-log_10_ scaled) from not significant (blue) to highly significant (red). Intracellular (n = 14) and extracellular (n = 48) proteins with Bonferroni’s corrected α = 3.8 × 10^−5^ (0.05/1305) are indicated on the plots.

Figure 2 shows scatter plots comparing concentration of 1,305 proteins in TNFα-stimulated HUVECs in hyperglycemia versus hyperglycemia alone in intracellular (Fig. 2a) and extracellular (Fig. 2b) HUVECs fractions. The values plotted are the mean RFU values (log_2_ scaled for 3 replicates) for TNFα-stimulated HUVECs in hyperglycemia (*y*-axis) versus hyperglycemia (*x*-axis) alone groups. The color of each point indicates the *p* values intensity (−log_10_ scaled) from highly significant proteins (red dots) to non-significant proteins (blue dots). Fourteen proteins were found to be differentially expressed (Bonferroni’s corrected α = 3.8 × 10^−5^ for 1305 proteins measured) in intracellular HUVECs, whereas 48 proteins were found to exhibit a significant variation following TNFα stimulation in the presence of hyperglycemia in the extracellular HUVECs. These significant proteins are marked on the scatterplots (Fig. 2a,b), and unmarked dot points indicate proteins that were unaffected by exposure to hyperglycemia and TNF-α treatment.

TNFα, used as our internal control, is one of the 1305 proteins measured on the SOMAscan assay. In the intracellular fraction of TNFα-stimulated HUVECs in the presence of hyperglycemia, the mean RFU value was 934 compared to 790 in the control HUVECs (fold change = 1.2, *p* = 5.9 × 10^−5^). In the extracellular space, the RFU signal was massively elevated, mainly due to the addition of TNFα into the culture media, compared with that of control HUVECs samples (Mean RFU: 20,970 vs. 302; fold change = 69.5, *p* = 5.1 × 10^−8^).

### Comparison of differentially expressed intracellular and extracellular proteins in TNFα-stimulated HUVECs and hyperglycemia

**Figure 3:**
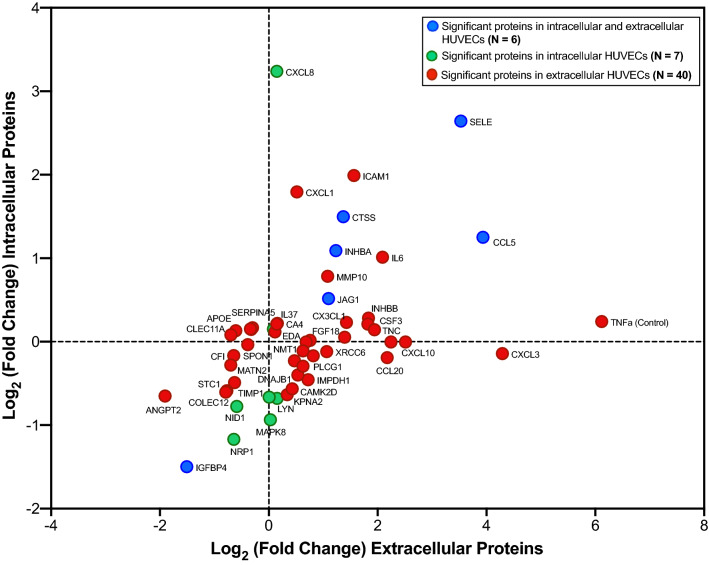
A scatterplot showing fold changes of significant proteins in intracellular vs. extracellular HUVECs subjected to TNFα treatment in hyperglycemia condition. Threshold for the significance used: α = 3.8 × 10^−5^ (Bonferroni’s correction for 1305 proteins measured on the SOMAscan platform). Fold change is a ratio of a mean concentration of a protein in HUVECs incubated with TNFα in hyperglycemia condition to a mean concentration of a protein in HUVECs cultured in hyperglycemia alone condition.

A scatterplot of the fold changes between differentially expressed intracellular (n = 14) versus extracellular (n = 48) proteins (excluding significant KRIS proteins) subjected to hyperglycemia and TNFα treatment is shown in Fig. 3. Subsequent plot filtering uncovered 6 significant proteins in both intracellular and extracellular HUVECs, 7 significant proteins only in intracellular HUVECs and 40 significant proteins only in extracellular HUVECs, cultured in hyperglycemia and TNFα treatment relative to expression in HUVECs with hyperglycemia alone condition (Fig. 3). The detailed list of 53 differentially expressed proteins and their corresponding intracellular and extracellular protein levels (expressed as fold changes) are shown in Table 2.

**Table 2:** List of 53 differentially expressed proteins and their corresponding intracellular and extracellular protein levels (expressed as fold changes) following TNFα treatment in hyperglycemia condition versus hyperglycemia alone.

Protein name	Gene symbol	Intracellular		Extracellular	
		Fold change	*p* value	Fold change	*p* value
**Significant intracellular and extracellular (n = 6)**					
C–C motif chemokine 5	CCL5	2.38	3.3 × 10^−5^	15.3	5.0 × 10^−6^
Cathepsin S	CTSS	2.82	2.7 × 10^−5^	2.58	1.8 × 10^−5^
Insulin-like growth factor-binding protein 4	IGFBP4	0.35	6.2 × 10^−6^	0.35	2.0 × 10^−5^
Inhibin beta A chain	INHBA	2.13	3.2 × 10^−5^	2.35	9.4 × 10^−7^
Protein jagged-1	JAG1	1.43	1.0 × 10^−5^	2.14	2.2 × 10^−6^
E-selectin	SELE	6.25	8.0 × 10^−6^	11.53	4.5 × 10^−7^
**Significant intracellular (n = 7)**					
Complement C1q subcomponent	C1QA/B/C	1.11	3.7 × 10^−5^	1.06	n.s
Interleukin-8	CXCL8	9.44	2.8 × 10^−5^	1.11	n.s
Tyrosine-protein kinase Lyn	LYN	0.62	1.3 × 10^−5^	1.11	n.s
Tyrosine-protein kinase Lyn, isoform B	LYN	0.63	7.0 × 10^−6^	1.00	n.s
Mitogen-activated protein kinase 8	MAPK8	0.52	1.2 × 10^−5^	1.02	n.s
Nidogen-1	NID1	0.58	7.0 × 10^−6^	0.67	n.s
Neuropilin-1	NRP1	0.44	1.6 × 10^−5^	0.64	n.s
**Significant extracellular (n = 40)**					
Angiopoietin-2	ANGPT2	0.64	n.s	0.27	1.1 × 10^−5^
Apolipoprotein E (isoform E4)	APOE	1.11	n.s	0.79	3.0 × 10^−5^
Carbonic anhydrase 4	CA4	1.16	n.s	1.11	2.1 × 10^−5^
Calcium/calmodulin-dependent protein kinase type II subunit delta	CAMK2D	0.68	n.s	1.35	2.3 × 10^−5^
C–C motif chemokine 20	CCL20	0.88	n.s	4.52	4.2 × 10^−7^
Complement factor I	CFI	0.89	n.s	0.64	8.1 × 10^−6^
Stem cell growth factor-beta	CLEC11A	1.09	n.s	0.66	9.5 × 10^−6^
Stem cell growth factor-alpha	CLEC11A	1.06	n.s	0.62	2.9 × 10^−5^
Collectin-12	COLEC12	0.66	n.s	0.58	5.1 × 10^−6^
Granulocyte colony-stimulating factor	CSF3	1.16	n.s	3.53	1.7 × 10^−5^
Cystatin-SA	CST2	1.11	n.s	0.8	5.1 × 10^−6^
Fractalkine	CX3CL1	1.17	n.s	2.69	2.0 × 10^−5^
Growth-regulated alpha protein	CXCL1	3.47	n.s	1.43	8.4 × 10^−6^
C-X-C motif chemokine 10	CXCL10	1.00	n.s	5.70	8.4 × 10^−6^
Gro-beta/gamma	CXCL3/L2	0.91	n.s	19.59	1.8 × 10^−7^
C-X-C motif chemokine 5	CXCL5	1.00	n.s	1.61	2.1 × 10^−6^
DnaJ homolog subfamily B member 1	DNAJB1	0.76	n.s	1.45	1.1 × 10^−5^
Ectodysplasin-A, secreted form	EDA	1.08	n.s	1.08	3.4 × 10^−5^
Fibroblast growth factor 18	FGF18	1.04	n.s	2.63	1.0 × 10^−6^
Heterogeneous nuclear ribonucleoprotein A/B	HNRNPAB	0.89	n.s	1.76	6.2 × 10^−6^
Intercellular adhesion molecule 1	ICAM1	3.97	n.s	2.96	1.2 × 10^−5^
Interleukin-37	IL37	1.16	n.s	1.11	1.7 × 10^−5^
Interleukin-6	IL6	2.02	n.s	4.25	1.9 × 10^−5^
Inosine-5′-monophosphate dehydrogenase 1	IMPDH1	0.73	n.s	1.65	4.1 × 10^−6^
Inhibin beta A:Inhibin beta B chain	INHBA/BB	1.22	n.s	3.56	3.7 × 10^−7^
Importin subunit alpha-1	KPNA2	0.64	n.s	1.26	1.0 × 10^−5^
Matrilin-2	MATN2	0.82	n.s	0.62	1.2 × 10^−5^
Stromelysin-2	MMP10	1.72	n.s	2.12	4.8 × 10^−6^
Glycylpeptide N-tetradecanoyltransferase 1	NMT1	0.93	n.s	1.55	7.0 × 10^−6^
Urokinase-type plasminogen activator	PLAU	1.00	n.s	4.74	5.6 × 10^−6^
1-phosphatidylinositol 4,5-bisphosphate phosphodiesterase gamma-1	PLCG1	0.85	n.s	1.39	1.6 × 10^−5^
Plasma serine protease inhibitor	SERPINA5	1.12	n.s	0.81	9.9 × 10^−6^
Spondin-1	SPON1	0.97	n.s	0.76	3.2 × 10^−5^
Stanniocalcin-1	STC1	0.66	n.s	0.59	2.5 × 10^−5^
Metalloproteinase inhibitor 1	TIMP1	0.71	n.s	0.65	1.4 × 10^−5^
Tenascin	TNC	1.10	n.s	3.84	2.3 × 10^−6^
Tumor necrosis factor (internal control)	TNF	1.18	n.s	69.46	5.1 × 10^−8^
TNF-inducible gene 6 protein	TNFAIP6	1.01	n.s	1.69	7.2 × 10^−6^
Polyubiquitin K48-linked	UBB	0.82	n.s	1.55	1.6 × 10^−5^
X-ray repair cross-complementing protein 6	XRCC6	0.92	n.s	2.09	2.7 × 10^−5^

n.s., not significant. Threshold for the significance used: α = 3.8 × 10^−5^ (Bonferroni’s correction for 1305 proteins measured on the SOMAscan platform). Fold change is a ratio of a mean RFU concentration of a protein in HUVECs incubated with TNFα in hyperglycemia to a mean RFU concentration of a protein in HUVECs cultured in hyperglycemia alone condition.

### Classification of proteins differentially expressed in intracellular and extracellular of TNFa-stimulated HUVECs and hyperglycemia

**Figure 4:**
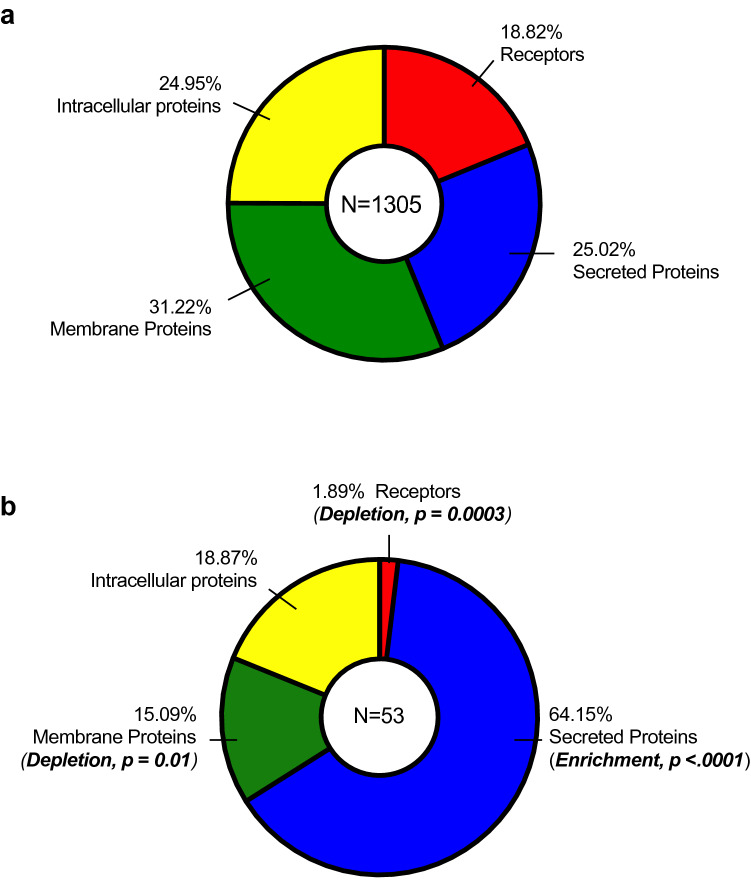
Protein classifications on SOMAscan. Classifications of (**a**) all proteins included on the SOMAscan platform and (**b**) proteins differentially expressed in intracellular and extracellular fractions of hyperglycemia and TNFα-stimulated HUVECs. Enrichment or depletion of certain protein classes was conducted using two-sided Fisher’s exact tests over a background of 1305 proteins.

The SOMAmer reagents were selected for 1,305 proteins. The proteins could be grouped into 4 classes that included receptors (19%), secreted proteins (25%), membrane proteins (31%) or intracellular proteins (25%) (Fig. 4a). The differentially expressed proteins listed in Table 2 comprised of 2% receptors, 64% secreted proteins, 15% membrane proteins and 19% intracellular proteins (Fig. 4b). In comparison with the distribution of these proteins in the total SOMAscan set, there was a notable abundance of secreted proteins (enrichment, *p* < 0.0001), whereas there were significant depletions of receptors (*p* = 0.0003) and membrane (*p* = 0.01) proteins. We observed no significant enrichment or depletion for intracellular proteins (Fig. 4b).

### Functional enrichment analysis of differentially expressed proteins

**Figure 5:**
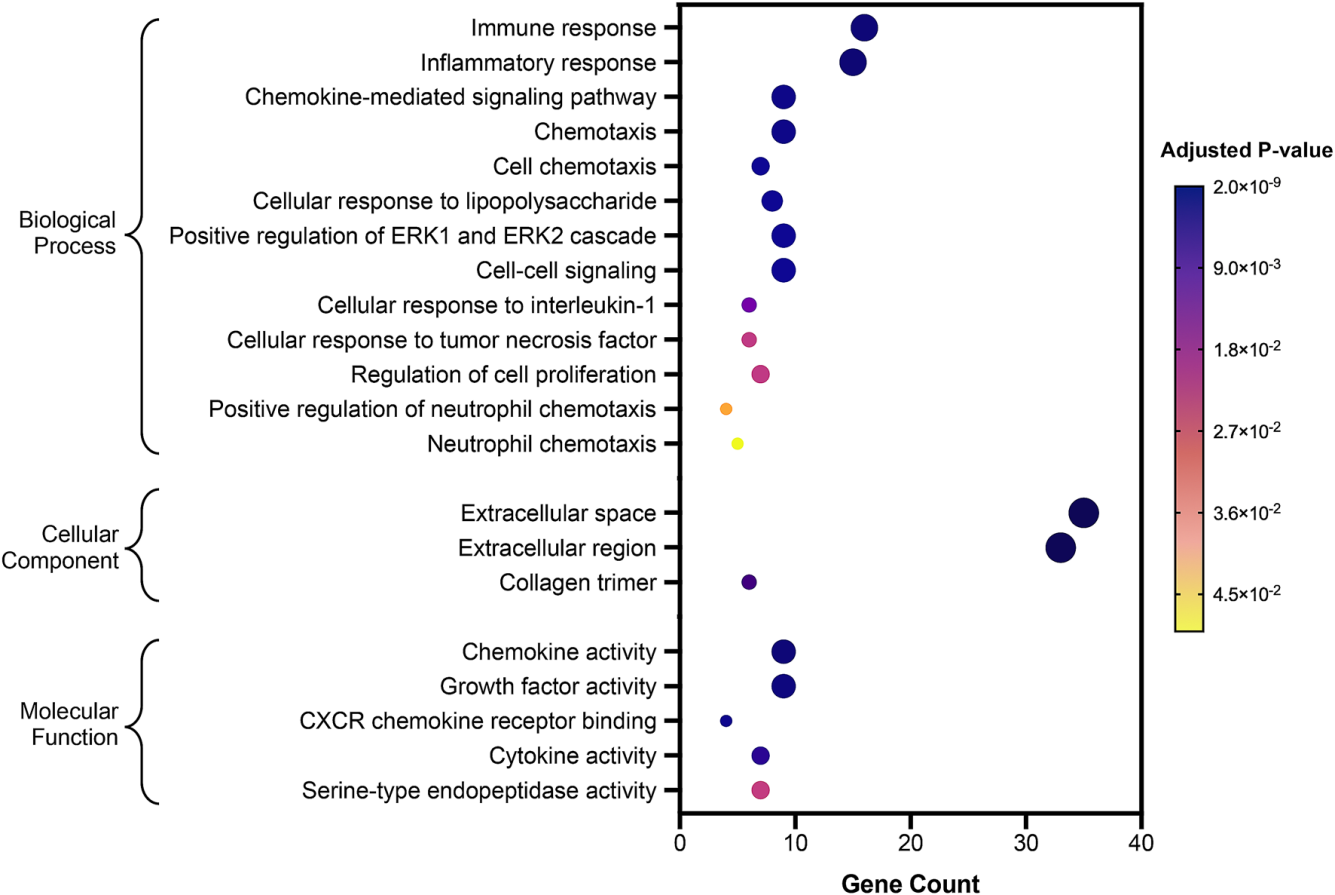
Functional enrichment analysis of differentially expressed proteins. Results of over-representation (or enrichment) analysis using DAVID Bioinformatics showing the gene ontology (GO) terms that were significantly enriched in GO Biological Process, Cellular Component and Molecular Function. The largest gene counts are plotted in order of gene count. The size of the dots represents the number of proteins in the significant protein list associated with the GO terms and the color of the dots represents the adjusted *p* values.

To analyze the biological context of differentially expressed proteins in intracellular and extracellular of TNFα-stimulated HUVECs in the presence of hyperglycemia, the list of 53 significant proteins (Fig. 4b, excluding KRIS proteins) were used as input for functional enrichment [over-representation of gene ontology (GO)] analysis using DAVID Bioinformatics database. Figure 5 summarizes the GO classification terms (biological processes, cellular component and molecular function) that have been linked with the proteins found to be differentially expressed in TNFα-stimulated HUVECs in high glucose versus high glucose alone. Biological processes that have been linked with these proteins include immune and inflammatory responses, chemokine signaling pathway, cell chemotaxis, cellular response to TNF and regulation of cell proliferation (Fig. 5). The GO cellular component displayed an enrichment of the extracellular space and the extracellular region (Fig. 5). In addition, chemokine activity, growth factor activity and cytokine activity were also found to be enriched (GO molecular function).

## Discussion

It has been hypothesized that elevated levels TNFα in presence of hyperglycemia might be important mechanisms that underlie the development of DKD^9^. Our recent study found a set of 17 circulating KRIS proteins that were strongly associated with progression to ESKD^17^, In this in vitro HUVECs study, we examined whether intracellular and extracellular levels of the KRIS proteins were regulated by exposure to high levels of TNFα and hyperglycemia. Levels of KRIS proteins as well as 1300 other proteins were measured using the SOMAscan proteomics platform. Overall, the levels of the KRIS proteins were not altered in intracellular or extracellular fractions of TNFα and hyperglycemia stimulated HUVECs. However, HUVECs exposed to these conditions showed significant up-regulation of a distinct set of 53 proteins (mainly in extracellular fraction). Circulating levels of these proteins were not associated with progression to ESKD in diabetes in our previous study^17^.

The present study is a first report to provide a complete global proteomic profile of TNFα-stimulated HUVECs in the presence of hyperglycemia with special emphasis on the investigation of the extracellular/secreted matrix proteome, considered of high importance in DKD. In parallel to the supernatants, we also examined the intracellular/cell lysate fraction, corresponding to the path of proteins on their way to be secreted into circulation. In addition, this study presents the first comparative global analysis of intracellular versus extracellular proteomes of TNFα-stimulated HUVECs in the presence of hyperglycemia fractionated by cellular location (intracellular and extracellular spaces).

Inflammatory processes play an essential role in the pathophysiology of DKD and other diabetes complications^14,26,27^. We recently reported circulating plasma levels of TNF receptors (including TNFR1 and TNFR2,) and other inflammatory proteins as an extremely robust and independent predictors of risk of ESKD in DKD^17^. The current study demonstrated that although TNFα protein level was extremely high in the extracellular HUVECs fraction, elevated TNFα level did not up-regulate extracellular levels of the KRIS proteins. Surprisingly, TNFR1 was the most significantly down-regulated protein in extracellular HUVECs, whereas TNFR2 protein levels were unaffected following TNFα treatment and hyperglycemia.

There is limited evidence regarding regulation of intra- and extra-cellular levels of TNF receptors and the other KRIS proteins. Several explanations were proposed. One postulates the role of TNF-converting enzyme (TACE, also named ADAM17) sheddase. This is a primary sheddase and/or activator of TNFα and TNF family receptors, leading to the proteolytic cleavage and release of ectodomains into the extracellular space^21,22,28^. Another mechanism for the generation of the soluble TNF receptors included the constitutive production of TNFR1 within exosome-like vesicles^29^. Hawari et al. reported that the major soluble form of TNFR1 is a full-length 55-kDa protein in human serum and lung epithelial lining fluid, whereas supernatants from human vascular endothelial cells contain only the full-length 55-kDa TNFR1^29^. In the present study, we have not determined whether circulating TNFR proteins measured in HUVECs were either fully cleaved or uncleaved or a combination of both forms. Regardless of the postulated mechanisms, the results of our study demonstrated that exposure to TNFα and hyperglycemia did not impact any of these mechanisms in HUVECs.

TNFα and its receptors, TNFR1 and TNFR2, constitute a complex signaling network, with both TNFα receptors differently activated by membrane and soluble TNFα. Their biological interactions are complex. TNFα signaling through these receptors induces cellular responses ranging from the production of pro-inflammatory cytokines to the stimulation of cellular proliferation, differentiation and cell migration as well as the initiation of cell death or apoptosis^30,31^. Interestingly, although we cannot exclude the impact of TNFa signaling through TNFR1 and TNFR2 on other proteins, our study showed that it did not result in increased production (intracellular levels) and secretion (extracellular levels) of KRIS proteins. Furthermore, the biological processes that were enriched with proteins stimulated by TNFa do not include apoptotic processes, one of the major pathways that is activated through TNFR1/2.

The present study also aimed to determine whether there are better protein signatures that distinguish between TNFα-stimulated HUVECs in the presence of high glucose versus high glucose alone conditions. Our global proteomic analysis indicated that TNFα induces/regulates the expression of many other proteins involved in immune response, chemokine and cytokine activities, and inflammatory processes. Among differentially expressed proteins, we observed several well-known cell membrane proteins that were involved in leukocyte recognition and recruitment including ICAM-1, and E-selectin, which were previously identified to be up-regulated upon inflammatory response of endothelial cells after TNFα stimulation^6,8^. Chemokines/cytokines are known to be pro-inflammatory and can be triggered during immune response to attract immune cells to the site of inflammation, whereas others are involved during normal tissue growth and development^32^. Taken together, our present findings reveal that HUVECs exposed to a hyperglycemic and TNFα environment regulates a distinct set of proteins, not associated with DKD and progression to ESKD.

Different mechanisms of regulation by TNFa of the distinct set of 53 proteins in HUVECs may be considered. Interestingly, several proteins that were intracellularly down-regulated in response to elevated glucose and TNFα environment were found to be up-regulated or in excess in extracellular HUVECs. How do the right proteins get to the right places or, more specifically, how do cells decide which proteins to retain and which ones to secrete to the cell exterior? This may be simply based on the protein abundance in the cells or perhaps other specific sorting signals direct proteins to different parts of the cell or they get exported out of the cell and into the extracellular space. When a protein is made, it will either remain in the cytosol or enter the endoplasmic reticulum (ER) during translation if they have an amino sequence called a signal peptide, which is a series of hydrophobic regions generally found near the N-terminus of the protein that facilitates the penetration and transport through the ER membrane^33,34^. This signal peptide along with other signals decide the final destinations, including residence in the ER (retention signal), lysosomes or the plasma membrane (stop-transfer signal) or getting exported to the cell exterior^35^. Other possible pathways of protein secretion were also proposed. First, proteins might be directly transported to the plasma membrane; second, accumulation of proteins underneath specific regions of the plasma membrane might be secreted to the cell exterior as a result of membrane blebbing; and third, the formation of tiny vesicles inside the cell, called exosomes, but are then released and broken in the extracellular space^36^.

Several limitations should be considered when interpreting our study findings. The present study is limited by a relatively low number of replicates and the reliance on a single cell type (HUVECs) and the results of this study need to be replicated and expanded using other cells as a target for TNFα. This study is, however, novel in several aspects. Firstly, we further confirmed our previous observations in DKD that TNFα ligand effects were negligible on TNF receptors, and secondly, the use of highly multiplexed SOMAscan platform, and thirdly, we performed the first global proteomic analysis directly comparing the intracellular and the extracellular/secreted matrix proteome, in TNFα-stimulated HUVECs exposed to a hyperglycemic condition, allowing for a more detailed picture of the inflammatory processes in fraction-specific components.

## Methods

### Comparisons of KRIS expression levels using in vitro cell culture approaches

To determine the best model system to study the effect of TNFα stimulation under high glucose conditions, we assessed the expression levels of KRIS in the cell lysate and supernatant in 3 human cell lines; umbilical vein endothelial cells (HUVECs), renal proximal tubule epithelial cells (RPTECs), and fibroblasts. HUVEC cell strain was randomly selected from among 62 cell strains recently used in our study^37,38^, RPTECs (CRL-4031) were purchased from the American Type Culture Collection (USA) and cultured according to the manufacturer’s protocol, and skin fibroblasts were obtained from a patient with type 1 diabetes and cultured as previously described^39^. The expression levels of KRIS in the cell lysates and supernatants from the 3 human cell lines were determined using the custom-made Olink proteomics assay. This high-throughput proteomic platform relies on two specific probes (dual recognition) through Olink’s proprietary Proximity Extension Assay (PEA) technology^40^. The cell lysates and supernatants from 3 human cell lines were processed at the Olink Bioscience laboratory of Olink Bioscience (Uppsala, Sweden).

### Preparation of human umbilical vein endothelial cells (HUVECs) and cell culture

Human Umbilical Vein Endothelial Cells (HUVECs) were isolated from umbilical cords of 62 newborns delivered by healthy Caucasian mothers between the 36th and the 40th gestational week at the Hospital of Chieti and Pescara in Italy^37^. The umbilical cords were obtained at the time of delivery when a cesarean section was performed. Due to privacy issues, we do not have any detailed information regarding the pregnant women’s healthy history except that they were healthy with no history of diabetes or any cardiovascular complications. A study that used these cell strains was recently published^38^. For the present study, one HUVEC cell strain out of 62 HUVEC strains was selected randomly.

Protocols to collect HUVEC strain were in agreement with the ethical standards of the local Institutional Committee on Human Experimentation (Reference Number: 1879/09COET) and with the Declaration of Helsinki Principles. The protocol was approved by the Institutional Review Board and the participating subjects signed the informed consent. The Joslin Diabetes Center Committee on Human Studies approved the experimental procedures for this study.

The details of the HUVECs culture preparation are described elsewhere^38^. For this study, HUVECs were grown to sub-confluence, then 150,000 cells/well were plated in 6 well plates. Following 24 hours (h), cells were serum-starved (0.5% FBS) and cultured in 4.5 g/L D-glucose (high glucose) or incubated for 24 h with TNFα (10 ng/mL) in high glucose. TNFα at a concentration of 10 ng/mL was selected in this study because it is considered the optimal functional concentration of TNFα in many cell types and in experimental studies^41,42^. It was shown that after the first 24 h, TNFα was active at the start of the experiments and its activity started to decline after 24 h. Then, supernatants were collected in tubes, centrifuged a 14,000 rpm for 5 min (min) to clarify before stored a − 80 °C. Cells were trypsinized, transferred to a 50 mL polypropylene tube, and centrifuged at 1200 rpm for 10 min. Supernatants were discarded and cell pellets gently re-suspended in phosphate buffer saline (PBS) and transferred to pre-labeled 2.0 mL polypropylene tubes. Cell suspensions were centrifuged and all supernatants carefully removed. Cell pellets were quickly snap frozen in liquid nitrogen and immediately stored at − 80 °C.

### Preparation of total cell lysate

Cell pellet was re-suspended in 200 μL Mammalian Protein Extraction Reagent (M-PER™, lysis buffer) and 1X HALT protease inhibitor (ThermoFisher), per kit instructions. The lysed cells were centrifuged at 14,000× g for 5 min and the clarified lysate was collected. The total protein amount was quantified using the BCA Protein Assay kit (ThermoFisher) and 2.4 μg of proteins were used in the SOMAscan assay.

### The SOMAscan proteomic assay

The SOMAscan proteomic platform uses single-stranded DNA aptamers and the platform is facilitated by a new generation of the Slow Off-rate Modified Aptamer (SOMAmer) reagents that benefit from the aptamer technology developed over the past 20 years^43,44^. The SOMAscan platform offers a remarkably dynamic range, and this large dynamic range results from the detection range of each SOMAmer reagent in combination with three serial dilutions of the sample of interest: the 40% (the most concentrated sample), 1% and 0.005% (the least concentrated sample) dilution groups to detect low, medium and high abundant proteins, respectively. The assay readout is reported in relative fluorescent units (RFU) and is directly proportional to the target protein amount in the original sample. The details of the SOMAscan proteomics platform are described elsewhere^23,24^.

### Statistical analysis

All statistical analyses were performed using SAS for Windows, version 9.4 (SAS Institute, Cary, NC). SOMAscan RFU values were log_10_-transformed to stabilize the variance prior to analysis. Fold change is a ratio of a mean RFU concentration of a protein in HUVECs cultured in TNFα in high glucose condition to a mean RFU concentration of a protein in HUVECs cultured in high glucose. Statistical significance of protein expression level changes between TNFα-stimulated and control HUVECs was determined by the paired two-tailed Student’s t-test and differentially expressed proteins at the Bonferroni’s correction α = 2.9 × 10^−3^ (17 KRIS proteins) and α = 3.8 × 10^−5^ (1305 proteins measured on the SOMAscan platform) were considered statistically significant. Enrichment or depletion of certain protein classes was conducted using two-sided Fisher’s exact tests over a background of 1305 proteins. Functional enrichment analyses were performed using DAVID Bioinformatics database^45,46^.

## Supplementary Information

Supplementary Information.

## Data Availability

The datasets generated and/or analyzed during the current study are available from the corresponding author upon reasonable request.
