# Supplementary material for: Effect of TNFα stimulation on expression of kidney risk inflammatory proteins in human umbilical vein endothelial cells cultured in hyperglycemia
Source: Sci Rep. 2021 May 27;11:11133. doi: 10.1038/s41598-021-90496-w (PMC8160214; doi:10.1038/s41598-021-90496-w)
Supplement: Supplementary file 1 — Supplementary Information. [file 41598_2021_90496_MOESM1_ESM.pdf]

Supplementary Information

Effect of TNFα Stimulation on Expression of Kidney Risk Inflammatory Proteins in Human Umbilical Vein Endothelial Cells Cultured in Hyperglycemia

Zaipul I. Md Dom[1,2], Caterina Pipino[1,2,3], Bozena Krolewski[1,2], Kristina O'Neil[1], Eiichiro Satake[1,2] and Andrzej S. Krolewski[1,2,*]

[1]Research Division, Joslin Diabetes Center, Boston, MA, USA; [2]Department of Medicine, Harvard Medical School, Boston, MA, USA; [3]Department of Medical, Oral and Biotechnological Sciences, Center for Advanced Studies and Technology, University G. d'Annunzio of Chieti-Pescara, Chieti, Italy

Table S1. List of the 17 circulating KRIS proteins associated with risk of development of ESKD and their corresponding extracellular and intracellular protein levels in HUVECs following TNFα treatment in hyperglycemia condition. TNFα, tumor necrosis factor alpha; TNF-RSF, tumor necrosis factor receptor superfamily. Data presented as mean relative fluorescence unit (RFU).

**Protein Name**	**Gene Symbol**	**Intracellular**	**Extracellular**
		**Hyperglycemia + TNFα**	**Hyperglycemia + TNFα**
TNF-RSF Members			
Tumor necrosis factor receptor superfamily member 1A	TNFR1	1133.48	823.03
Tumor necrosis factor receptor superfamily member 1B	TNFR2	9318.73	2200.81
Tumor necrosis factor receptor superfamily member 21	TNFRSF21	1259.75	8111.74
Tumor necrosis factor receptor superfamily member 19	TNFRSF19	262.99	130.16
Tumor necrosis factor receptor superfamily member 27	EDA2R	3741.12	689.33
Tumor necrosis factor receptor superfamily member 19L	RELT	643.93	381.77
Other Proteins			
Interleukin-15 receptor subunit alpha	IL15RA	1841.55	357.48
Interleukin-17F	IL17F	821.99	197.67
Complement decay-accelerating factor	CD55	9842.67	3003.05
CMRF35-like molecule 6	CD300C	990.71	397.20
Tumor necrosis factor ligand superfamily member 15	TNFSF15	927.94	8209.81
C-C motif chemokine 14	CCL14	100.20	146.43
C-C motif chemokine 15	CCL15	1685.95	257.69
Macrophage colony-stimulating factor 1	CSF1	7576.03	2134.95
Hepatitis A virus cellular receptor 2	HAVCR2	1106.82	275.30
Interleukin-1 receptor type 1	IL1R1	191.54	69.87
Interleukin-18 receptor 1	IL18R1	8196.60	1205.96

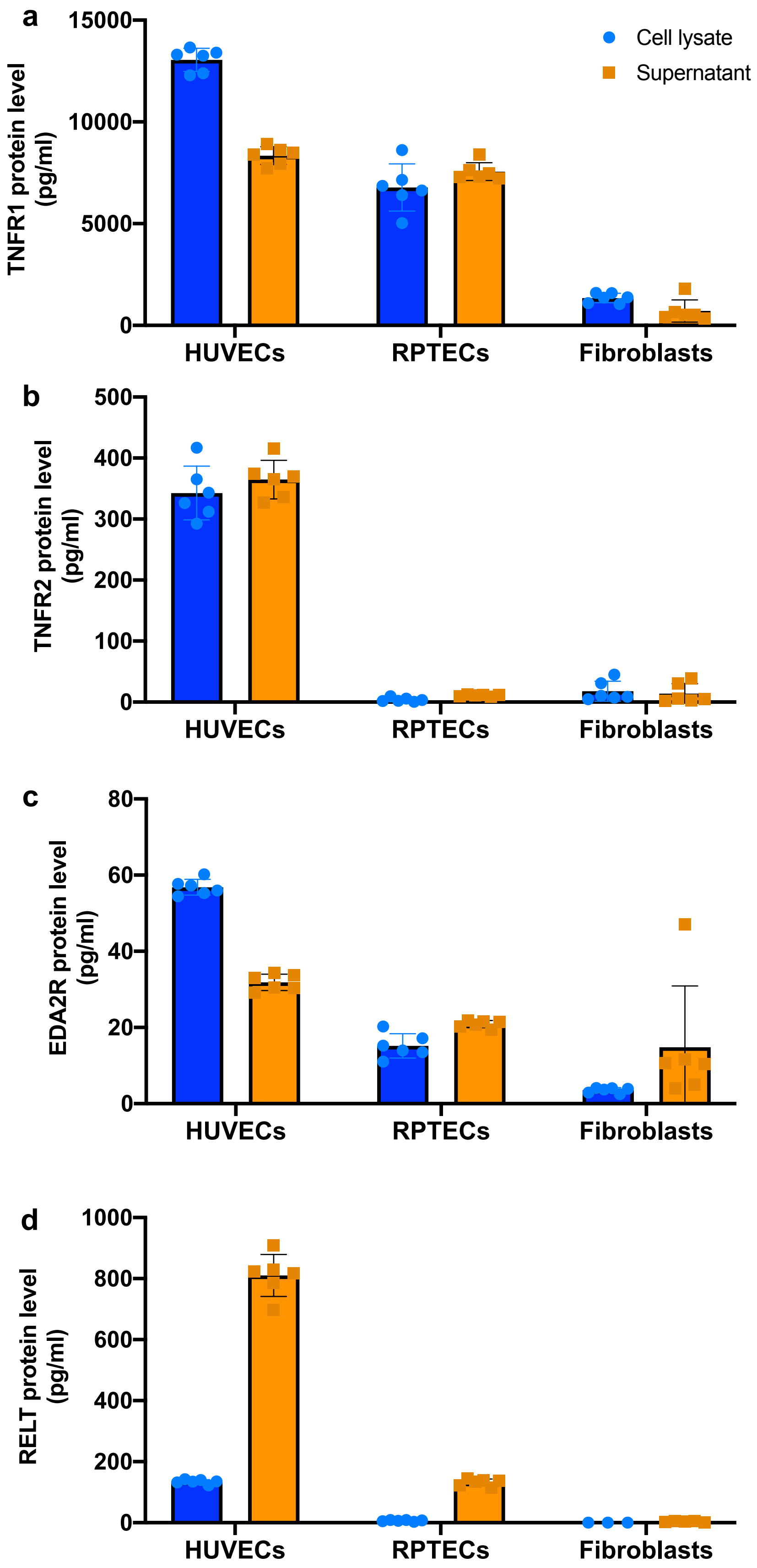

a
TNFR1 protein level (pg/ml)
15000
10000
5000
0
Cell lysate
Supernatant
HUVECs
RPTECs
Fibroblasts
b
TNFR2 protein level (pg/ml)
500
400
300
200
100
0
HUVECs
RPTECs
Fibroblasts
c
EDA2R protein level (pg/ml)
80
60
40
20
0
HUVECs
RPTECs
Fibroblasts
d
RELT protein level (pg/ml)
1000
800
600
400
200
0
HUVECs
RPTECs
Fibroblasts

Figure S1. Expression protein levels (pg/ml) of select KRIS proteins, **(A)** TNFR1, **(B)** TNFR2, **(C)** EDA2R and **(D)** RELT in 3 human cell lines (HUVECs, Renal Proximal Tubule Epithelial Cells (RPTECs) and Fibroblasts) measured using the custom-made Olink platform.

a

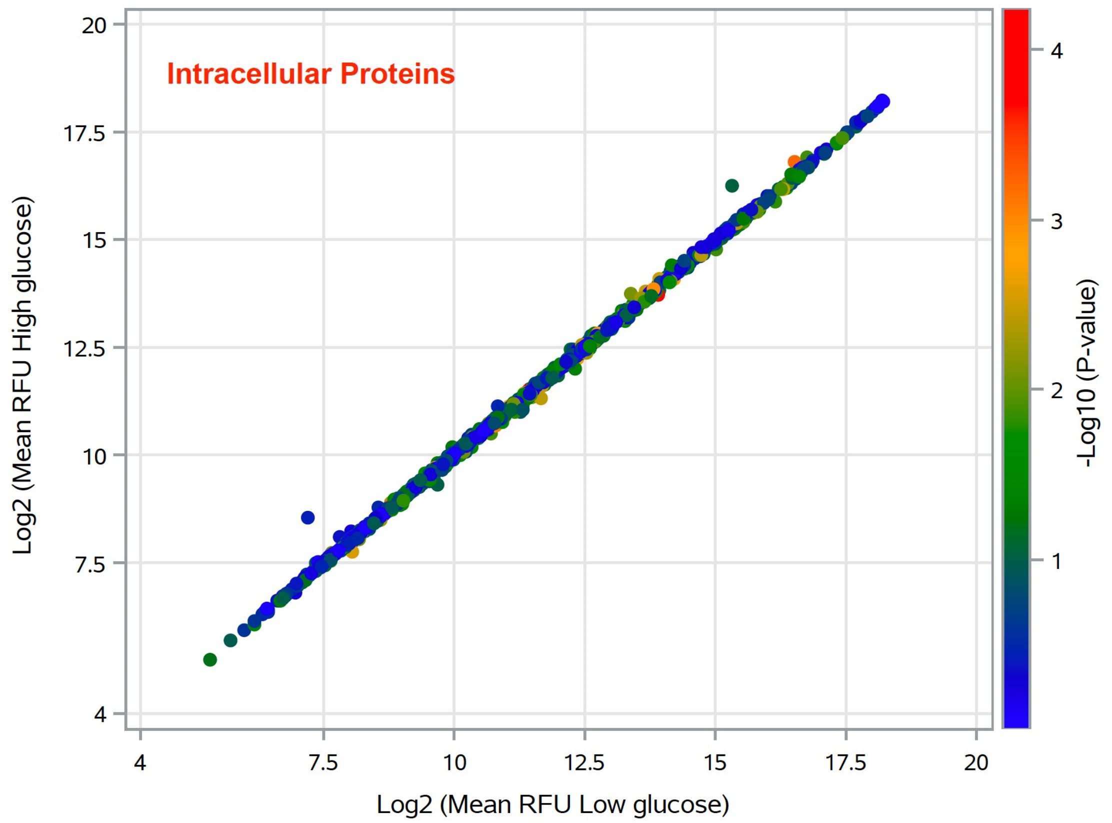
Intracellular Proteins
Log2 (Mean RFU High glucose)
Log2 (Mean RFU Low glucose)
-Log10 (P-value)
20
17.5
15
12.5
10
7.5
4
4
7.5
10
12.5
15
17.5
20
4
3
2
1

b

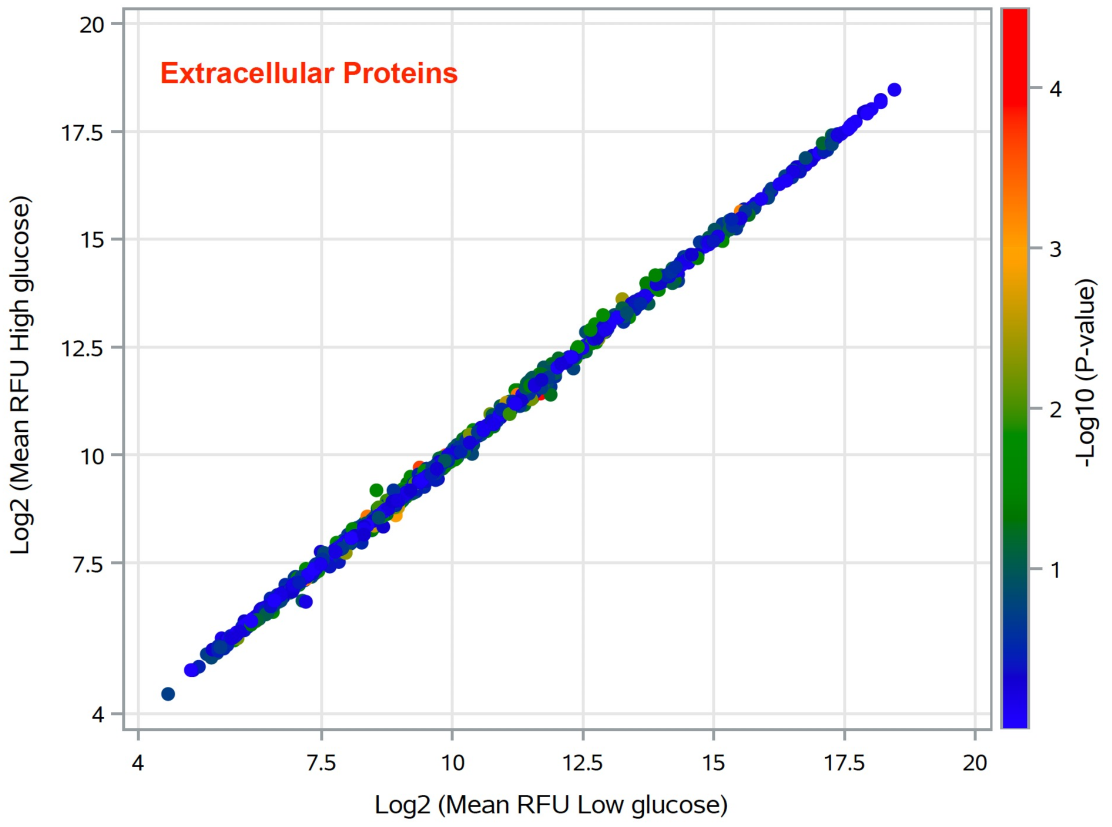
Extracellular Proteins
Log2 (Mean RFU High glucose)
Log2 (Mean RFU Low glucose)
-Log10 (P-value)
20
17.5
15
12.5
10
7.5
4
4
7.5
10
12.5
15
17.5
20
4
3
2
1

Figure S2. Scatterplots comparing **(A)** intracellular and **(B)** extracellular protein expression profiles in HUVECs exposed to high glucose (4.5 g/L) vs. low glucose (1.0 g/L) alone. The values plotted are the mean RFU values (log_2 scaled for 3 replicates) for the high glucose (*y* axis) and the low glucose (*x* axis) groups. The color of each point indicates the P-values intensity (-log_{10} scaled) from not significant (blue) to highly significant (red).
